# Contralateral risk-reducing local therapy in breast cancer patients with* BRCA1/2* mutations: systemic review and meta-analysis

**DOI:** 10.1186/s12935-021-02194-2

**Published:** 2021-09-25

**Authors:** Ziqi Jia, Jiaxin Li, Yuelun Zhang, Xin Wang, Jiahua Xing, Zeyu Xing, Xin Huang, Gang Liu, Menglu Zhang, Kexin Feng, Jiang Wu, Wenyan Wang, Jie Wang, Jiaqi Liu, Xiang Wang

**Affiliations:** 1 Department of Breast Surgical Oncology, National Cancer Center/National Clinical Research Center for Cancer/Cancer Hospital, Chinese Academy of Medical Sciences and Peking Union Medical College, 100021 Beijing, China; 2 School of Clinical Medicine, Chinese Academy of Medical Sciences & Peking Union Medical College, Beijing, 100005 China; 3 Medical Research Center, Peking Union Medical College Hospital, Peking Union Medical College and Chinese Academy of Medical Sciences, Beijing, 100730 China; 4 School of Medicine, Nankai University, Tianjin, 300071 China; 5 Department of Breast Surgery, Peking Union Medical College Hospital, Peking Union Medical College and Chinese Academy of Medical Sciences, Beijing, 100730 China; 6 Department of Breast Surgery, Beijing Tiantan Hospital, Capital Medical University, Beijing, 100070 China; 7 Department of Ultrasound, National Cancer Center/National Clinical Research Center for Cancer/Cancer Hospital, Chinese Academy of Medical Sciences and Peking Union Medical College, Beijing, 100021 China

**Keywords:** *BRCA1*, *BRCA2*, Contralateral risk-reducing mastectomy, Contralateral prophylactic irradiation

## Abstract

**Background:**

Unilateral breast cancer (UBC) patients with germline pathogenic *BRCA1/2* variants have a higher risk of developing contralateral breast cancer (CBC) and need contralateral risk-reducing local treatments, including contralateral risk-reducing mastectomy (CRRM) and prophylactic irradiation (CPI). The aim of our study was to systematically explore the efficacy of CRRM and CPI in reducing CBC risk and increasing survival.

**Methods:**

A search was done, and eligible randomized trials and cohort studies should include and compare UBC patients with germline pathogenic *BRCA1/2* variants who have and have not received contralateral risk-reducing local treatment. Random-effects meta-analysis was used in this study. Primary outcomes of the studies included overall survival (OS) and the incidence of contralateral breast cancer (CBC), and secondary outcomes included breast cancer-specific survival (BCSS).

**Results:**

A total of five studies with 1769 UBC patients with germline pathogenic *BRCA1/2* variants were enrolled in our meta-analysis. CRRM was correlated with a lower risk of CBC in UBC patients with germline pathogenic *BRCA1/2* variants (summary RR = 0.07; 95%CI 0.03–0.13, I^2^ = 3%), a significantly increased OS (summary RR, 1.15; 95%CI 1.04–1.26, I^2^ = 26%) and a significantly increased BCSS (summary RR, 1.18; 95%CI 1.07–1.31, I^2^ = 64%) compared with surveillance. CPI also decreased the risk of CBC (RR 0.02; 95%CI 0.05–0.88) but did not significantly improve OS (RR 0.97; 95%CI 0.90–1.05) and BCSS (RR 0.97; 95%CI 0.90–1.05) compared with surveillance.

**Conclusions:**

CRRM reduces CBC risk and increases OS and BCSS in UBC patients with germline pathogenic *BRCA1/2* variants, and could be offered as a risk-reducing local treatment. For those who oppose CRRM, CPI could be offered for CBC-risk reduction, while its survival benefit is still uncertain.

**Supplementary Information:**

The online version contains supplementary material available at 10.1186/s12935-021-02194-2.

## Background

Breast cancer has become the most commonly diagnosed cancer since 2020 [1], 0.85–3.0% of whom carry a germline pathogenic *BRCA*1/2 variation [2–5]. Unilateral breast cancer (UBC) patients harboring *BRCA1/2* mutations have higher risks of developing contralateral breast cancer (CBC) after the diagnosis of the first breast cancer (1st BC) than those who do not. The 10-year CBC risk after the diagnosis of the 1st BC was reported as 18–25%, 32–42%, and 15.5–23.8% in non-Jewish Caucasian, Jewish, and Asian women, respectively, as compared to only 3–7% in non-carriers [6–8]. Contralateral risk-reducing mastectomy (CRRM) was offered to UBC patients with *BRCA1/2* mutations historically to reduce CBC risk. In the pathological review of CRRM surgical specimens of *BRCA1/2 *mutation carriers, the risk of CBC lifted even higher because about 11% of occult cancer was observed despite the negative pre-surgical imaging findings [9]. A gradually increasing number of UBC patients carrying *BRCA1/2 *mutations are adopting CRRM, especially in those under the age of 45 considering these patients have more a more aggressive tumor biology and higher expectations for a longer survival [10–12]. However, the efficacy of CRRM needed evaluation.

Previous meta-analysis studies agreed that CRRM reduced the risk of CBC in UBC patients with high familial/genetic risk (RR 0.04; 95%CI 0.02–0.09;* P* < 0.001) [13], especially those harboring a *BRCA1/2 *mutation (reduced by 91–93%; RR 0.07; 95%CI 0.04–0.15; P = 0.34) [14, 15]. However, there has been conflicting evidence on whether CRRM improves overall survival (OS) and breast cancer-specific survival (BCSS) in UBC patients harboring *BRCA1/2 *mutations [10, 16–20]. Meta-analyses focusing on the survival benefit of CRRM revealed no significant difference in BCSS (HR 0.78; 95% CI 0.44–1.39; P = 0.40) by Valachis et al., and a significantly decreased all-cause mortality rate (HR 0.51; 95% CI 0.368–0.714) by Li et al. [14, 15]. Based on the current evidence of reduction in CBC risk and all-cause mortality, recent guidelines from National Comprehensive Cancer Network (NCCN) recommended that CRRM should be offered as a choice to patients with a *BRCA1/2 *mutation and diagnosed with UBC according to formal consensus [21].

Under the overall trend towards less invasive oncologic care, the de-escalation of CRRM and non-invasive substitute of CRRM have been explored. Nevertheless, risk-reducing local treatment is still needed because systemic treatments such as risk-reducing bilateral salpingo-oophorectomy (BSO), although effective (HR, 0.44; 95% CI 0.21–0.91; P = 0.03), may not be considered by women who have not yet given birth, and adjuvant chemotherapy (HR 1.03; 95%CI 0.68–1.55; P = 0.90), or tamoxifen (HR, 0.59; 95% CI 0.35–1.01; P = 0.05), cannot significantly reduce metachronous CBC risk [7, 22]. Contralateral prophylactic irradiation (CPI) has recently been proposed as a potential alternative to CRRM with advantages such as being non-disfiguring and less invasive [23]. Recently, Evron et al. reported the beneficial results of a phase-II clinical trial of CPI in early-stage UBC patients with *BRCA1/2 *mutations [24].

Here, we performed a systemic review and meta-analysis of the contralateral risk-reducing local treatments, including CRRM and CPI, in female UBC patients harboring *BRCA1/2* mutations. We aimed to update the summarized efficacy of CRRM on CBC risk reduction and survival improvement, while compare the efficacy of CPI with CRRM, in order to provide further preventive oncological care suggestions for *BRCA1/2* carriers.

## Methods

### Protocol and registration

This systemic review is reported according to the Preferred Reporting Items for Systematic Reviews and Meta-Analyses (PRISMA) statement [25]. The protocol for this systematic review was registered on PROSPERO (CRD42020199036) and is available in full from: https://www.crd.york.ac.uk/prospero/display_record.php?RecordID=199036. since Aug 5, 2020 [26].

### Eligibility criteria

Eligible studies were those that included and compared UBC patients harboring *BRCA1/2* mutation who had received and had not received contralateral risk-reducing local treatment. Primary surgery of UBC include breast-conserving surgery and mastectomy. Contralateral risk-reducing local treatment is defined as modified radical or radical mastectomy or irradiation on the contralateral breast. The outcomes of the studies include CBC and OS, and secondary outcome include BCSS. Follow-up time was not an exclusion criteria and comprehensive search including randomized controlled trials and observational studies was performed. Exclusion criteria include: (1) patients for whom contralateral breast cancer incidence in terms of hazard ratio or risk ratio is not available will be excluded; (2) patients with *BRCA1/2* mutation having any other type of cancer (except breast cancer); (3) reviews and clinical studies with designs other than retrospective observational, prospective observational cohort studies, and interventional studies were excluded from the analysis. Conference abstracts were included in the qualitative systemic review but not the quantitative synthesis because they were not peer-reviewed and may include unreported bias.

### Information source and search strategy

A search was done on Aug 14, 2020 in the following databases: MEDLINE via PubMed, Web of Science, EMBASE, Scopus, Cochrane Central Register of Controlled Trials, Cochrane Database of Systemic Reviews, and ClinicalTrials.gov. Search terms included the keywords: breast neoplasms, *BRCA*, contralateral AND mastectomy, contralateral AND radiotherapy, survival, contralateral breast cancer and their synonyms and Medical Subject Headings (MeSHs). The complete searching strategy is shown in Additional file 1: Methods S1, S2. We also hand-searched references cited by the included research articles and found five studies that met the inclusion criteria.

### Study selection, data items and collection process

Titles and keywords were assessed after deduplication, and abstracts and full-texts were obtained and evaluated to determine whether the study met the eligibility criteria. Eligibility assessment and data extraction were completed independently by two researchers and disagreements were resolved through discussion with a third researcher. Effect measures were extracted as component values (i.e., incidence in both groups) for the local treatment and control groups. When such data was not accessable, researchers were contacted for additional unreported data. For each outcome, study-specific risk ratios (RRs), or hazard ratios (HRs), and 95% confidence intervals (CIs) were collected for CBC risk and survival for patients receiving CRRM or CPI versus surveillance. When necessary, HRs were calculated using data extracted from graphical format (e.g. Kaplan–Meier curve).

### Quality assessment

Cochrane risk-of-bias tool for randomized trials should be used for included randomized trials. And ROBINS-I [27], a tool to assess the risks of bias of non-randomized studies recommended by Cochrane Handbook for Systemic Reviews of Intervenions, was used if cohort studies were included [28]. This tool required the review authors to describe a ‘target trial’, which is a hypothetical pragmatic randomized trial of the interventions compared in the study, conducted on the same participant group without features putting it at risk of bias and need not to be feasible or ethical. Signaling questions were structured according to a fixed set of domains of bias, including confounding, selection bias, information bias, reporting bias, etc. Based on answers to the signaling questions, judgements for each bias domain, and for overall risk of bias, can be ‘Low’, ‘Moderate’, ‘Serious’ or ‘Critical’ risk of bias. No conflict of interests that might influence author judgements were involved. A study can be assessed at low, moderate, serious, and critical risk of bias.

### Summary measures and synthesis of results

We performed an updated meta-analysis of CRRM in UBC patients carrying *BRCA1/2 *mutations. Basic demographic and clinical characteristics were summarized and compared between CRRM and CPI studies. Pooled rates along with 95% CIs for CBC risk, OS, and BCSS were calculated as dichotomous data. RR was selected as the primary meta-analytic measure of association due to the lack of time-to-event data in certain individual studies. Because HR and 95% CIs for BCSS were not reported in a study [17], estimates were obtained from the Kaplan-Meier curve with data extracted by Engauge-Digitizer software [29].

Statistical heterogeneity was tested across trials by the Cochran’s Q test. A two-sided α value of less than 0.1 was taken to indicate between-trial heterogeneity, with is represented by I^2 ^values (25%-low, 50%-moderate, 75%-high level of heterogeneity) [30]. Random-effects meta-analysis (the DerSimonian and Laird method) of RR was used. Two-sided *P*< 0.05 was considered statistically significant for the synthesis of the primary and secondary outcomes. All statistical analyses were conducted using Stata version MP 16.0 (StataCorp, TX) and forrest plots and included data were generated using Review Manager 5.4 (The Nordic Cochrane Centre) [31].

## Results

### Study selection

**Fig. 1:**
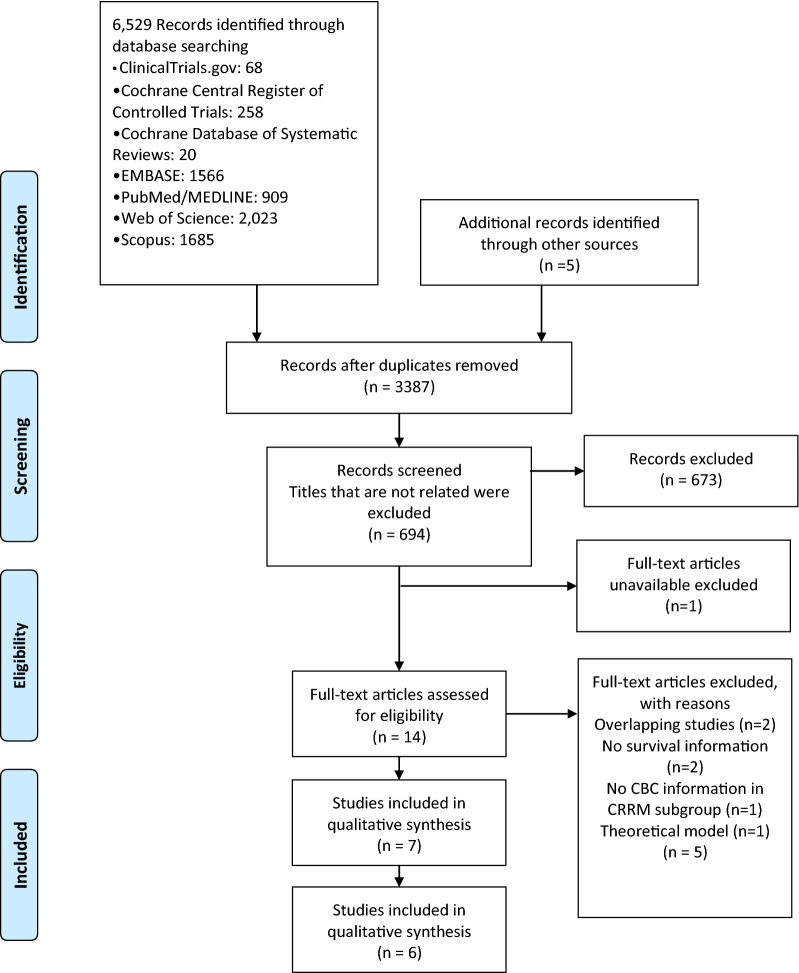
PRISMA flowchart of literature search for contralateral local therapy in unilateral breast cancer patients with germline pathogenic *BRCA1/2* mutations. *CBC* contralateral breast cancer, *CRRM* contralateral risk-reducing mastectomy

The initial literature search yielded 6529 records (Fig. 1). A non-randomized clinical trial, which studied the efficacy of CRRM in Japanese UBC patients carrying *BRCA1/2 *mutations, was still recruiting and thus excluded from this study [32]. Of the 14 studies with full texts after selection and deduplication, six were excluded according to the eligibility criteria described in the meta-analysis protocol: inappropriate study design, intervention, population, or outcomes [33]. Two of the studies were conducted on the same population [7, 18], and the most recently published one was included [18]. Two studies were not designed to study CRRM efficacy in *BRCA* carriers, but included patients who underwent CRRM and were harboring *BRCA1/2*[16, 34]. In total, seven articles [10, 11, 17−19, 35, 36] were included in the qualitative analysis and the results of five studies [10, 11, 17−19, 36] were pooled in a meta-analysis.

### Study characteristics

The characteristics of included studies for quality assessment are presented in Table 1. Four of the studies were observational retrospective cohort studies and the other three were prospective cohort studies. A total of 3087 UBC patients carrying *BRCA1/2* mutations were included in this systemic review, with 1706 included in the meta-analysis. Characteristics of patients of the individual studies included in the qualitative systemic review are shown in Table 2. Six of the studies [10, 11, 17−19, 35] focused on the efficacy of CRRM and the remaining study [36] focused on CPI. Sample sizes of the included studies ranged from 88 to 1018 participants. Follow-up years of individual studies ranged from 3.5 to 14.3 years. Six of the seven studies were multicenter studies [10, 11, 16−19, 36]. One study has been reported as a conference abstract because not all observational endpoints have been achieved and thus, was excluded from quantitative analysis [35]. Among the included studies, five studies reported CBC as primary of secondary outcome measure. OS is reported as a hazard ratio in four studies [10, 17−19] and as a risk ratio in three studies [11, 35, 36] .

**Table 1:** Characteristics of studies included in the qualitative systemic review

First author	Year	Country	Participants	Race/ethnicity	Study design	Year range	Treatment	ROBINS-I^d^	Follow-up (PT/no PT)(years)	Primary outcome in the original study
van Sprundel [17]	2005	The Netherlands (M)	148 *BRCA1/2* carriers	NR	RC	~ 2003	CRRM	Moderate	Mean 3.5 (3.4/3.1)	CBC, BCSS, OS, DMR
Kiely [11]	2009	Australia (M)^a^	1018 *BRCA1/2* carriers from high-risk families	Caucasian 95% Asian 1% Other 2% Unknown 2%	RC	1997–2008	CRRM	Serious	Median 11.1 (8/11.7)	OS, CBC
Evans [10]	2013	The UK (M)	698 *BRCA1/2* carriers	NR	PC	1985–2010	CRRM	Moderate	Median 8.8/7.3	CBC, OS
Metcalfe [18]	2014	Canada (M)	390 patients^c^	NR	RC	1975–2008	CRRM	Moderate	Median 9.7/8.6	CBC, OS
Heemskerk-Gerritsen [19]	2015	The Netherlands (M)^b^	583 *BRCA1/2* carriers	NR	PC	1980–2011	CRRM	Moderate	Median 14.3	CBC, OS
Fachinetti [35]	2019	Italy (S)	88 *BRCA1/2* carriers	NR	RC	2006–2016	CRRM	NA	Median 11.4 (11.4/11.3)	CBC, OS
Evron [36]	2019	Isreal (M)	162 *BRCA1/2* carriers	NR	PC	2008–2017	CPI	Moderate	Median 10	CBC, OS

*RC* retrospective cohort study, *PC* prospective cohort study, *CBC* contralateral breast cancer, *BCSS* breast-cancer specific survival, *OS* overall survival, *DMR* distant metastatic recurrence, *M* multicenter study, *S* single-center study, *NR* Not reported, *NA* Not applicable

^a^Patients were enrolled in the research program called kConFab (Kathleen Cuningham Foundation Consortium for Research into Familial Breast Cancer)

^b^Members of breast and/or ovarian cancer centers in the HEBON study (nationwide Dutch study on risk assessment and gene–environment interactions)

^c^336 patients were proved to be *BRCA1/2* carriers, 54 were not tested but from family with a *BRCA* mutation

^d^Specific assessment for each signal question in the ROBINS-I tool see: Additional file 1: Method S2

**Table 2:** Characteristics of patients of individual studies included in the qualitative systemic review

First author	Year	*BRCA1/BRCA2*	No. with PT (*BRCA1/2*)	No. with no PT (*BRCA1/2*)	Mean age at UBC diagnosis (PT/NPT)	Mean age at PT	Stage	Interval
van Sprundel [17]	2005	115/43	79(60/19)	69(55/14)	38.0/39.4	41.9	I-III	4.0 ± 0.5
Kiely [11]	2009	161/127	154	864	46.7	NR	I-III	NR
Evans [10]	2013	NR	105(51/54)	593 (NR)	40.3/44.5	44	I-III	1.1(0.0-13.3)
Evans [10] (matched)^a^	2013	102/108	105(51/54)	105(51/54)	40.3/41.5	44	I-III	1.1(0.0-13.3)
Metcalfe [18]	2014	226/158	181(103/76)	209(123/82)	43.6/41.3	NR	I-II	2.3
Heemskerk- Gerritsen [19]	2015	454/129	242(193/49)	341(261/80)	38/42	NR	I-III	2.0(0-20.2)
Fachinetti [35]	2019	70/58^b^	NR	NR	44	NR	NR	NR
Evron [36]	2019	81/81	54/29	59/22	NR	50.4	I-III	NR

Interval: Time between primary BC diagnosis and prophylactic treatment

*PT* prophylactic treatment, *UBC* unilateral breast cancer

^a^105 patients of the surveillence group were matched for the contralateral prophylactic mastectomy group and formed a prospective cohort

^b^Not all patient received surgery

### Risk of bias within studies and confounding factors

Due to ethical and operational reasons, none of the eligible studies were randomized [10, 11, 17−19, 35, 36]. Risk of bias within studies was assessed by the ROBINS-I tool [27] (Table 1). Confounding factors should be carefully discussed and considered in the analysis. It was reported that the 5-year cumulative risk of CBC is 13% in UBC patients harboring *BRCA1* mutation and 8% in those with *BRCA2 *mutation. And the cumulative risk of CBC at 10-years is 40 and 26%, respectively [37]. Thus, the mutated gene should be considered as a confounding factor, and subgroup analysis should as well be performed. Younger age at 1st BC diagnosis, higher Body Mass Index (BMI), lobular histology, greater histological grade and size of the tumor, ER/PR-negative status, and higher breast density are all variables that raise CBC risk to a lesser extent. Furthermore, these variables were also less favorable to a longer OS and BCSS. A combination of these characteristics might be associated with an even further increase in risk [33, 38, 39]. Individual inclination was also observed in relationship with younger age at diagnosis, lobular histology, ER/PR-positive pathological status, and Caucasian race, which might act as confounding factors as well [40]. However, the meta-analysis-included studies did not provide according subgroup analysis in how CRRM or CPI reduces the risk of CBC and prolongs OS or BCSS in these subgroups. Three of the studies are prospective cohort studies that had moderate risk of bias and can be considered as a sound non-randomized study. The other four studies were retrospective cohort studies in which two were of moderate risk of bias, one was of serious risk of bias, and one was of no information of bias.

### Contralateral breast cancer risk

**Fig. 2:**
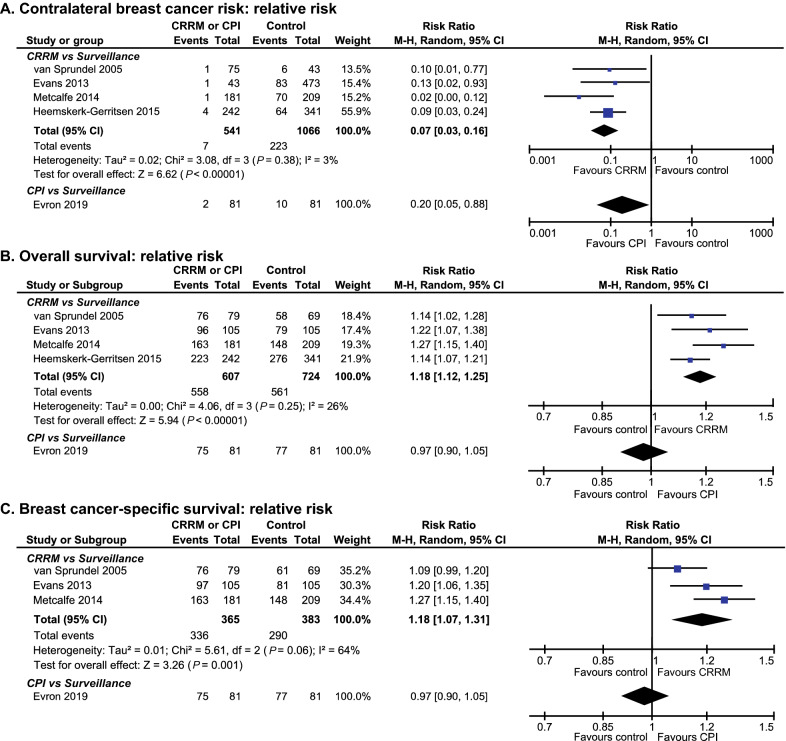
Contralateral breast cancer: relative risk. **A** CRRM, CPI, and Contralateral breast cancer risk in unilateral breast cancer patients carrying *BRCA1/2* mutation. **B** CRRM, CPI, and overall survival in unilateral breast cancer patients carrying *BRCA1/2* mutation. **C** CRRM, CPI, and breast cancer-specific survival in unilateral breast cancer patients carrying *BRCA1/2* mutation. The width of the horizontal line represents the 95% CI of the individual study, and the square proportional represents the weight of each study. The weight was calculated by the sample size of each individual study, and was presented by the percentage of total. The diamond represents the pooled RR and 95% CI. *CBC* contralateral breast cancer, *CRRM* contralateral risk-reducing mastectomy, *CPI* contralateral prophylactic irradiation

For CRRM, four non-overlapping studies of low to moderate risk of bias investigated the CBC risk in UBC patients carrying *BRCA1/2 *mutations who were treated with CRRM relative to those who did not receive CRRM [10, 17−19]. In total, 1607 UBC patients carrying *BRCA1/2* mutations were included in the meta-analysis. Results showed that CRRM was correlated with a lower risk of CBC in UBC patients carrying *BRCA1/2* mutations (summary RR, 0.07; 95%CI 0.03–0.13; Fig. 2A). As reported by Heemskerk-Gerritsen et al., most metachronous CBCs in *BRCA1/2* carriers had a favorable tumour stage, with 87% having a Tis/T1 classification, and 79% having a node-negative disease. Nevertheless, the HR/HER2 status of CBC is similar to the 1st BC, with 73% being triple-negative. The risk of CBC is slightly different between *BRCA1* and *BRCA2*, being 13 and 8%, respectively (P = 0.122). For CPI, Evron et al. reported two cases (2.5%) of CBC among 81 patients who received CPI and 10 cases (12.4%) of CBC among 81 patients who did not receive CPI and underwent surveillance (RR 0.20; 95%CI 0.05–0.88; Fig. 2A) [36]. No study has looked at the risk decrease related with CPM in *BRCA1* and *BRCA2* mutation carriers individually.

### Overall survival

For CRRM, four non-overlapping studies of low to moderate risk of bias investigated OS in UBC patients carrying *BRCA1/2 *mutations who were treated with CRRM relative to those who did not receive CRRM [10, 17−19]. In total, 724 UBC patients carrying *BRCA1/2* mutations were included in the meta-analysis. Results show that CRRM was associated with a significantly increased OS in UBC patients carrying *BRCA1/2* mutations (summary RR, 1.15; 95%CI 1.04–1.26; Fig. 2B). Four of the aforementioned studies analyzed OS as time-to-event data and also reported HR as an effect measure. When assessed according to HR, CRRM was also associated with a significantly increased OS in UBC patients carrying *BRCA1/2* mutations (summary HR, 0.53; 95%CI 0.38–0.74; Additional file 1: Figure S1). To circumvent confounding factors, Evans et al. reported a significanly improved OS (HR 0.37, 95%CI 0.17–0.8) in 105 unilateral breast cancer patients with germline *BRCA1/2* mutations who underwent CRRM were compared to 105 specifically matched controls, the OS improvement is still significant after adjusted for risk-reduction bilateral salpingo-oophorectomy (HR 0.43, 95%CI 0.2–0.95).

For CPI, Evron et al. reported 6 cases (7.4%) of death among 81 patients who received CPI and 4 cases (4.9%) of CBC among 81 patients who did not receive CPI and underwent surveillance (RR 0.97; 95%CI 0.90–1.05; Fig. 2B) [36].

### Breast cancer-specific survival

For CRRM, three non-overlapping studies investigated BCSS in UBC patients carrying *BRCA1/2 *mutations who were treated with CRRM relative to those who did not receive CRRM [10, 17, 18]. In total, 383 UBC patients carrying *BRCA1/2* mutations were included in the meta-analysis. CRRM was associated with a significantly increased BCSS in UBC patients carrying *BRCA1/2* mutations (summary RR, 1.18; 95%CI 1.07–1.31; Fig. 2 C). Metcalfe et al. explored extensively on BCSS according to the length of follow-up. They reported a hazard ratio for the women treated with CRRM compared with control group of 0.52 (0.29 to 0.93; P = 0.03) for 20-year of follow-up and 0.20 (0.05 to 0.89; P = 0.03) for the second decade of follow-up, adjusted for *BRCA* mutation, tumour size, nodal status, age at diagnosis, year of diagnosis, chemotherapy, radiotherapy, and oophorectomy.

For CPI, all of the above-mentioned deaths were caused by breast cancer. Thus, for BCSS, RR is also reported as 0.97 (95%CI 0.90–1.05; Fig. 2C) [36].

### Toxicity and side-effects

CRRM has twice the complication rate when compared to unilateral mastectomy, and is also associated with longer surgery time, higher cost, and longer hospital stays, regardless of whether or not reconstruction is done [41–45]. The risk of complication is approximately the same in the index or the contralateral breast. Such complications are classified into surgical, oncologic, and aesthetic/reconstruction according to the etiology. Surgical complications including bleeding, infection, skin/tissue flap necrosis/loss, anesthesia complications, and thrombosis often have an early onset. Taking mastectomy and reconstructive complications into account, the incidence of complication is about 40–64%, and about 52% of patients undergo at least one unanticipated surgery [46]. The risk of surgical complication cannot be emphasized enough in patients who smoke or with potential comorbidities, such as obesity, diabetes, or cardiac/pulmonary comorbidities. Oncologic side-effects refer to the likelihood of worse prognosis, as patients who receive CRRM may delay adjuvant therapy or refuse further radiotherapy [47]. From the aesthetic/reconstruction aspect, 20–30% of patients who received CRRM reported worse outcomes regarding appearance, pain, and sexuality than expected [48, 49]. Although symmetry is one of the critical motivations of CRRM, especially in patients with large or ptotic breast, they should be advised for other available symmetry procedures, especially when breast conservation is advocated for all eligible patients with or without neoadjuvant systemic treatments.

**Fig. 3:**
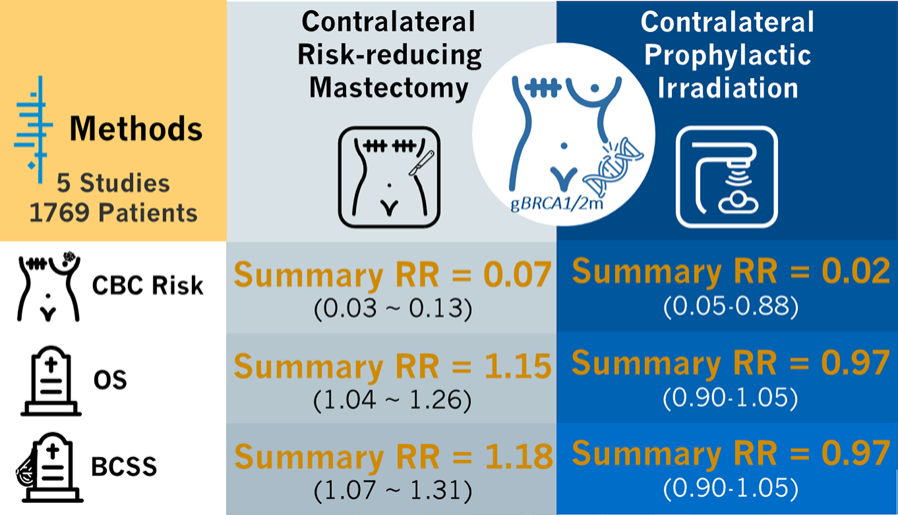
Summarizing figure of the meta-analysis comparing CRRM and CPI in *BRCA1/2* carriers. CBC, contralateral breast cancer; OS, overall survival; BCSS, breast-cancer specific survival; RR, risk ratio; g*BRCA1/2*m, germline *BRCA1/2* mutation

For patients who opted for CPI, the major concern was radiation-induced malignancy [50, 51]. In Evron et al.’s study, one case of pleomorphic sarcoma was reported and grade I–II acute irradiation toxicity was observed in most patients [36] (Fig. 3).

## Discussion

To the best of our knowledge, this is the first study that systemically reviews and compares contralateral risk-reducing local treatments, including CRRM and CPI, in *BRCA1/2* mutated UBC patients. We meta-analyzed the data for CRRM in CBC risk and mortality rates in UBC patients harboring *BRCA1/2* mutation, and compared it to the efficacy data of CPI (Fig. 3). Adverse events were also summarized and reported for these studies.

The results of the meta-analysis for CRRM suggested a 93% (95% CI 96–87%) reduction in CBC risk in UBC patients harboring *BRCA1/2* mutations who received CRRM. However, CRRM did not eliminate the risk of developing a secondary breast cancer in the residual contralateral breast. Additionally, our meta-analysis suggested an 18% increase in OS rate and an 18% increase in BCSS rate of UBC patients harboring *BRCA1/2* mutations who received CRRM. Although CRRM did not improve OS in UBC patients at average genetic risk [13], our study accorded with previous meta-analysis that CRRM can improve OS in UBC patients at elevated familial risk [13].

Based on the individual studies, guidelines from NCCN and European countries stated that CRRM should be offered as an option to UBC patients carrying *BRCA1/2 *mutations [21, 52–54]. No directly-relevant guidelines were published in Asian countries, likely due to the fact that no relevant data of Asian patients have been published. A non-randomized Japanese clinical trial is now under recruitment and may reveal the efficacy of CRRM in Asian population in the future [32]. However, the decision for CRRM is a personal decision affected by patients’ weigh on symmetry, anxiety during surveillance, and acceptance of removing a normal breast. Although post-surgical satisfaction is reported by most patients who received CRRM, 57.7% of *BRCA1/2* carriers forego the choice of CRRM before or after the surgery for primary breast cancer [55]. These *BRCA1/2* carriers, who refused CRRM, made this decision because their reluctance to remove a normal breast [56], but the risk of CBC is still worrying nonetheless.

CPI is a novel risk-reducing local intervention on the contralateral breast. Clinicians need to consider both the therapeutic effect of radiation to kill malignant and premalignant cells and the adverse effect of radiation to cause cancer [57]. Based on the fact that the genetic background is the same in both breasts, the risk reduction observed in the ipsilateral residual breast after adjuvant radiation results from radiation therapy [23]. This hypothesis was validated by an experimental breast cancer mouse model [58]. A phase II non-randomized clinical trial [36]and a study focusing on MRI images of breast cancer patients [59] were later published on the application of CPI in breast cancer patients. CPI is proposed as an alternative to UBC patients harboring *BRCA1/2* mutations who underwent standard loco-regional treatment for the primary breast cancer and declined CRRM. However, it is critical to understand the risk reduction efficacy of CRRM and CPI before further application. Compared to the summary efficacy of CRRM (summary RR, 0.07; 95%CI), CPI has a lower efficacy in CBC risk reduction (RR 0.20; 95%CI 0.05–0.13). In terms of OS and BCSS, the efficacy of CPI is less than CRRM, because CRRM significantly improved survival of UBC patients harboring *BRCA1/2* mutations while CPI did not. This conclusion might result from the relatively short follow-up period in the study of CPI. Heemskerk-Gerritson et al. reported that survival curves diverge between CRRM group and surveillance group after 10–11 years of follow-up [19], which is longer than the follow-up period of the CPI study (58 months). The reported average time from the diagnosis of the primary breast cancer to secondary primary breast cancer is 5.7 years [6]. However, a significant reduction in CBC risk is observed in the CPI study. We optimistically expect to see a more significant difference in CBC risk and a significant improvement in survival rate between patients who underwent CPI and surveillance in an updated report, as the reported trend in CRRM studies.

The study of CPI gathered patients who declined CRRM. The decision of using CRRM is influenced by many factors including age, family history, loco-regional surgery for 1st BC, etc [11]. Many of these factors are potential confounding factors in the survival analysis. Thus, comparison is made between the baseline demographic and clinical characteristics of the patients across the included studies. It is observed that patients who declined CRRM and opted for CPI are significantly older. This could also account for the insignificant result on OS and BCSS in the CPI study.

Nevertheless, our meta-analysis had several limitations. First, the study design of the included studies were not entirely accordant. Because of the invasive and irreversible nature of CRRM, it is infeasible to design a randomized controlled clinical trial. It is also not possible to randomize CPI either because of limited clinical study and untestified efficacy. To conduct a high-quality meta-analysis, studies that are well-designed and well-performed are needed. Therefore, we provide a proposed design for a non-randomized trial that is comparable to a well-performed randomized clinical trial on this topic (Additional file 1: Figure S1). This design considered the following domain as possible confounders: age at 1st BC diagnosis, year of 1st BC diagnosis, surgery for 1st BC, T stage, nodal involvement, grade, family history, involved gene: *BRCA1/BRCA2*. We also considered co-intervention as an important potential bias including: BSO, chemotherapy and endocrine therapy per standard guidelines, radiotherapy on the ipsilateral side. Also, our study could not perform subgroup analysis regarding *BRCA1/2* genes, age/ menopause status, ER/PR/HER2 status, or BMI, because the included studies did not report some of the clinical characteristics of their cohort, nor performed subgroup analyses. Our study referenced a limited number of studies; the referenced studies require long terms of follow-up and more studies are needed to be planned and conducted.

Breast cancer incidence is predicted to grow in the coming future, as global awareness of breast cancer screening is increasing and women lifestyle is shifting towards higher breast cancer risk [60, 61]. At the same time, the treatment regimen of breast cancer has significantly improved in the past few decades, resulting in lower mortality rates and a trend toward less invasive oncological care [62]. To facilitate such trend, a predictive risk score regarding the risk of CBC in hereditarily/genetically high risk patients is needed in the future to guide precision medicine tailored for individual patients.

## Conclusions

In conclusion, CPM and CPI are both available to be offered as an option to reduce CBC risk for UBC patients harboring *BRCA1/2* mutations. While, CPM is the only effective treatment with enough retrospective evidence to improve survival in this cohort until now. Under the trend toward less invasive oncological care, this systemic review and meta-analysis could provide helpful evidence-based guidance for clinicians when offering prophylactic treatment options to patients with *BRCA1/2* mutations. However, longer follow-up, larger sample size, and delicate pre-study protocols are still needed for both interventions, especially for CPI.

## Supplementary Information

**Additional file 1. **Searching strategy, quality assessment tool, and proposed design for a non-randomized trialSearching strategy, quality assessment tool, and proposed design for a non-randomized trial

## Data Availability

All data generated or analysed during this study are included in this published article and its additional information files.

## Abbreviations

UBC: Unilateral breast cancer
CBC: Contralateral breast cancer
CPM: Contralateral prophylactic mastectomy
CPI: Contralateral prophylactic irradiation
OS: Overall survival
CBC: Contralateral breast cancer
BCSS: Breast cancer-specific survival
1st BC: First breast cancer
NCCN: National Comprehensive Cancer Network
BSO: Bilateral salpingo-oophorectomy
PRISMA: Preferred Reporting Items for Systematic Reviews and Meta-Analyses
MeSHs: Medical Subject Headings
RR: Risk ratio
HR: Hazard ratios
CI: Confidence interval
