# Supplementary material for: Contralateral risk-reducing local therapy in breast cancer patients with BRCA1/2 mutations: systemic review and meta-analysis
Source: Cancer Cell Int. 2021 Sep 25;21:512. doi: 10.1186/s12935-021-02194-2 (PMC8466340; doi:10.1186/s12935-021-02194-2)
Supplement: Supplementary file 1 — Additional file 1. Searching strategy, quality assessment tool, and proposed design for a non-randomized trialSearching strategy, quality assessment tool, and proposed design for a non-randomized trial [file 12935_2021_2194_MOESM1_ESM.pdf]

Supplementary Online Content

Jia et al. Contralateral Risk-reducing Local Therapy in Unilateral Breast Cancer Patients with Germline Pathogenic BRCA1/2 Mutations: Systemic Review and Meta-analysis

eMethod 1. Searching Strategy of PubMed database for systemic review and meta-analysis of contralateral risk-reducing mastectomy and irradiation . . . . - 2 -

eMethod 2. ROBINS-I tool . . . . - 3 -

Specify a target randomized trial specific to the study. . . . . - 3 -

Specify a target randomized trial specific to the study. . . . . - 3 -

Specify the outcome that is being assess for risk of bias . . . . - 3 -

Specify the numerical result being assessed. . . . . - 3 -

Preliminary consideration of confounders . . . . - 5 -

Preliminary consideration of co-interventions . . . . - 5 -

Risk of bias assessment . . . . - 6 -

eFigure. Proposed design for a well-designed non-randomized trial that is comparable to a well-performed randomized trial . . . . - 13 -

References in the Supplement: . . . . - 14 -

This supplementary material has been provided by the authors to give readers additional information about their work.

eMethod 1. Searching Strategy of PubMed database for systemic review and meta-analysis of contralateral risk-reducing mastectomy and irradiation

("Breast Neoplasms"[Mesh] OR "Hereditary Breast and Ovarian Cancer Syndrome"[Mesh] OR “breast carcinomas” OR “breast carcinoma” OR "Breast Neoplasms" OR “Breast Neoplasm” OR “Breast Tumors” OR “Breast Tumor” OR “Mammary Carcinomas” OR “Mammary Carcinoma” OR “Mammary Neoplasm” OR “Mammary Neoplasms” OR “Breast Cancer” OR “breast cancers” OR “Cancer of the Breast” OR “Cancer of Breast” OR “Mammary Ductal Carcinomas” OR “Mammary Ductal Carcinoma” OR “Breast Invasive Ductal Carcinoma” OR “breast gland cancer” OR “breast gland neoplasm” OR “mamma cancer” OR “mammary cancer” OR “mammary gland cancer” OR “breast adenocarcinoma” OR “mammary adenocarcinoma” OR “breast carcinogenesis” OR “breast cancerogenesis” OR “mammary gland carcinogenesis” OR “breast carcinoma” OR “mamma carcinoma” OR "Hereditary Breast and Ovarian Cancer Syndrome" OR “HBOC Syndrome” OR “HBOC Syndromes” OR “BRCA1” OR “BRCA2”) AND ("prevention and control" [Subheading] OR "prevention and control" OR “preventive therapy” OR “prophylaxis” OR “preventive measures” OR “prevention” OR “control” OR “asepsis” OR “disease eradication” OR “protection” OR “prophylactic” OR “Prophylactically” OR “risk reduction” OR “risk reducing” OR “risk-reducing”) AND ("Mastectomy"[Mesh] OR "Mastectomy" OR “Mastectomies” OR “Mammectomy” OR “Mammectomies” OR “lumpectomy” OR “breast-conserving” OR “conserving surgery” OR “breast amputation” OR “breast resection” OR “Halsted operation” OR “irradiation” OR “radiotherapy” OR “radiation”) AND (“contralateral”) AND (“contralateral breast cancer” OR “CBC” OR “overall survival” OR "Mortality"[Mesh] OR "mortality" [Subheading] OR “Mortality” OR “Mortalities” OR “Case Fatality Rate” OR “Case Fatality Rates” OR “Death Rate” OR “Death Rates” OR “survival” OR “disease free” OR

“cancer free” OR “breast cancer specific” OR “distant metastasis free” OR “disease-free” OR “cancer-free” OR “breast-cancer specific” OR “distant-metastasis free” OR “asepsis” OR "Recurrence"[Mesh] OR "Recurrence" OR “Recurrences” OR “Relapse” OR “Relapses” OR “Recrudescence” OR “Recrudescences” OR “cancer recidive” OR “cancer regeneration” OR “breast cancer ”) NOT ("Animals"[Mesh] NOT ("Animals"[Mesh] AND "Humans"[Mesh]))

eMethod 2. ROBINS-I tool

Specify a target randomized trial specific to the study

Design	Individually randomized
Participants	Patients with breast cancer harboring *BRCA1* or *BRCA2* mutations
Experimental intervention	Contralateral risk-reducing mastectomy
Comparator	Not receiving any contralateral prophylactic loco-treatment.

Specify a target randomized trial specific to the study

The aim for this study is to assess the effect of starting and adhering to intervention: those who did not receive contralateral risk-reducing mastectomy were not included

Specify the outcome that is being assess for risk of bias

The risk of CBC: benefit of intervention

Overall survival: benefit of intervention

Breast cancer-specific survival: benefit of intervention

Specify the numerical result being assessed

The risk of CBC: RR (with 95%CI, P value), HR

Overall survival: RR (with 95%CI, P value), HR

Breast cancer-specific survival: RR (with 95%CI, P value), HR

Preliminary consideration of confounders

(i) Confounding domains listed in the review protocol			
Confounding domain	Measured variable(s)	Is there evidence that controlling for this variable was unnecessary?*	Is the confounding domain measured validly and reliably by this variable (or these variables)?
Age at 1st BC diagnosis	Age	No	Yes
Trend of CRRM becoming more prevalent	Year of 1st BC diagnosis	No	Yes
Surgery for 1st breast cancer	Mastectomy/Breast-conserving surgery	No	Yes
Tumor size	T stage	No	Yes
Nodal involvement	N stage	No	Yes
Mutation status	*BRCA1/BRCA2*	No	Yes

Preliminary consideration of co-interventions

(i) Co-interventions listed in the review protocol		
Co-intervention	Is there evidence that controlling for this co- intervention was unnecessary (e.g. because it was not administered)?	Is presence of this co-intervention likely to favour outcomes in the experimental intervention or the comparator
Bilateral oophorectomy	No	Favour experimental

(ii) Additional co-interventions relevant to the setting of this particular study, or which the study authors identified as important		
Co-intervention	Is there evidence that controlling for this co- intervention was unnecessary (e.g. because it was not administered)?	Is presence of this co-intervention likely to favour outcomes in the experimental intervention or the comparator
Chemotherapy	No	Favour experimental
Radiotherapy	No	Favour experimental
Hormonal therapy	No	Favour experimental

Risk of bias assessment

Study	Signalling questions and responding options								Risk of bias judgement
Bias due to confounding									
	1.1 Is there potential for confounding of the effect of intervention in this study?	1.2. Was the analysis based on splitting participants' follow up time according to intervention received? (If N/PN to 1.4-1.6 If Y/PY to 1.3)	1.3. Were intervention discontinuations or switches likely to be related to factors that are prognostic for the outcome? (If N/PN to 1.4-1.6 If Y/PY to 1.7-1.8)	1.4. Did the authors use an appropriate analysis method that controlled for all the important confounding domains? (If Y/PY to 1.5)	1.5. If Y/PY to 1.4: Were confounding domains that were controlled for measured validly and reliably by the variables available in this study?	1.6. Did the authors control for any postintervention variables that could have been affected by the intervention?	1.7. Did the authors use an appropriate analysis method that controlled for all the important confounding domains and for time-varying confounding? (If Y/PY to 1.8)	1.8. If Y/PY to 1.7: Were confounding domains that were controlled for measured validly and reliably by the variables available in this study?	
van Sprundel[1]	Y	N	-	Y	Y	N	-	-	Moderate
Kiely[2]	Y	N	-	Y	Y	N	-	-	Moderate
Evans[3]	Y	N	-	Y	Y	N	-	-	Moderate

Metcalfe[4]	Y	N	-	Y	Y	N	-	-	Moderate
Heemskerk-Gerritsen[5]	Y	Y	N	Y	Y	N	-	-	Moderate
Evron[6]	Y	N	-	Y	Y	N	-	-	Moderate

Bias in selection of participants into the study						
	2.1 Was selection of participants into the study (or into the analysis) based on participant characteristics observed after the start of intervention? If N/PN to 2.4	2.2 If Y/PY to 2.1: Were the post- intervention variables that influenced selection likely to be associated with intervention?	2.3 If Y/PY to 2.2: Were the post- intervention variables that influenced selection likely to be influenced by the outcome or a cause of the outcome?	2.4 Do start of follow-up and start of intervention coincide for most participants?	2.5 If Y/PY to 2.2 and 2.3, or N/PN to 2.4: Were adjustment techniques used that are likely to correct for the presence of selection biases?	
van Sprundel[1]	N	-	-	Y (For control group: date of mutation testing; for CRRM group: date of mutation testing or CRRM, whichever came last)	-	Low
Kiely[2]	N	-	-	NI	NI	NI
Evans[3] (matched)	N	-	-	Y (For control group:	-	Low

				start at the same interval from breast cancer as the length of time from the 1st BC to CRRM in the matched CRRM case;<br>For CRRM group: date of CRRM)		
Metcalfe[4]	N	-	-	Y<br>(For control group: from 1st BC diagnosis<br>For CRRM group: the date of CRRM surgery)	-	Low
Heemskerk-Gerritsen[5]	N	-	-	Y<br>(For control group: start at the same interval from breast cancer as the length of time from the 1st BC to CRRM in the matched CRRM case;<br>For CRRM group:	-	Low

				date of CRRM)		
Evron[6]	N	-	-	Y (For control group: the date of 1st BC diagnosis; For CPI group: the date of irradiation)	-	Low

Bias in classification of interventions				
	3.1 Were intervention groups clearly defined?	3.2 Was the information used to define intervention groups recorded at the start of the intervention?	3.3 Could classification of intervention status have been affected by knowledge of the outcome or risk of the outcome?	
van Sprundel[1]	Y	Y	N	Low
Kiely[2]	Y	Y	N	Low
Evans[3]	Y	Y	N	Low
Metcalfe[4]	Y	Y	N	Low
Heemskerk-Gerritsen[5]	Y	Y	N	Low
Evron[6]	Y	Y	N	Low

Bias due to deviations from intended interventions							
	to assess the effect of assignment to intervention		assess the effect of starting and adhering to intervention				
	4.1 Were there deviations from	4.2 If Y/PY to 4.1: Were	4.3 Were important co-interventions balanced	4.4 Was the intervention	4.5 Did study participants	4.6 If N/PN to 4.3, 4.4 or 4.5:	

	the intended intervention beyond what would be expected in usual practice?	these deviations from intended intervention unbalanced between groups and likely to have affected the outcome?	across intervention groups?	implemented successfully for most participants?	adhere to the assigned intervention regimen?	Was an appropriate analysis used to estimate the effect of starting and adhering to the intervention?	
van Sprundel[1]	-	-	N (BSO, surgery for 1st BC, chemotherapy, radiotherapy, endocrine therapy)	Y	Y	Y	Moderate
Kiely[2]	-	-	N (BSO, surgery for 1st BC, chemotherapy, radiotherapy, endocrine therapy)	Y	Y	N	Serious
Evans[3]	-	-	Y	Y	Y	-	Low
Metcalfe[4]	-	-	N (BSO, chemotherapy, endocrine therapy)	Y	Y	Y	Moderate
Heemskerk-Gerritsen[5]	-	-	N (BSO, surgery/chemotherapy/ endocrine therapy/ radiotherapy for 1st BC)	Y	Y	Y	Moderate
Evron[6]	-	-	Y	Y	Y	-	Low

Bias due to missing data						
	5.1 Were outcome data available for all, or nearly all, participants?	5.2 Were participants excluded due to missing data on intervention status?	5.3 Were participants excluded due to missing data on other variables needed for the analysis?	5.4 If PN/N to 5.1, or Y/PY to 5.2 or 5.3: Are the proportion of participants and reasons for missing data similar across interventions?	5.5 If PN/N to 5.1, or Y/PY to 5.2 or 5.3: Is there evidence that results were robust to the presence of missing data?	
van Sprundel[1]	Y	N	N	-	-	Low
Kiely[2]	N	N	Y	NI	NI	Serious
Evans[3]	Y	N	N	-	-	Low
Metcalfe[4]	Y	N	N	-	-	Low
Heemskerk-Gerritsen[5]	Y	N	N	-	-	Low
Evron[6]	Y	N	N	-	-	Low

Bias in measurement of outcomes					
	6.1 Could the outcome measure have been influenced by knowledge of the intervention received?	6.2 Were outcome assessors aware of the intervention received by study participants?	6.3 Were the methods of outcome assessment comparable across intervention groups?	6.4 Were any systematic errors in measurement of the outcome related to intervention received?	
van Sprundel[1]	N	Y	Y	N	Low
Kiely[2]	N	Y	Y	N	Low
Evans[3]	N	Y	Y	N	Low

Metcalfe[4]	N	Y	Y	N	Low
Heemskerk-Gerritsen[5]	N	Y	Y	N	Low
Evron[6]	N	Y	Y	N	Low

Bias in selection of the reported result				
	Is the reported effect estimate likely to be selected, on the basis of the results, from...			
	7.1 multiple outcome measurements within the outcome domain?	7.2 multiple analyses of the intervention-outcome relationship?	7.3 different subgroups?	
van Sprundel[1]	N	N	N	Low
Kiely[2]	N	N	N	Low
Evans[3]	N	N	N	Low
Metcalfe[4]	N	N	N	Low
Heemskerk-Gerritsen[5]	N	N	N	Low
Evron[6]	N	N	N	Low

eFigure. Proposed design for a well-designed non-randomized trial that is comparable to a well-performed randomized trial

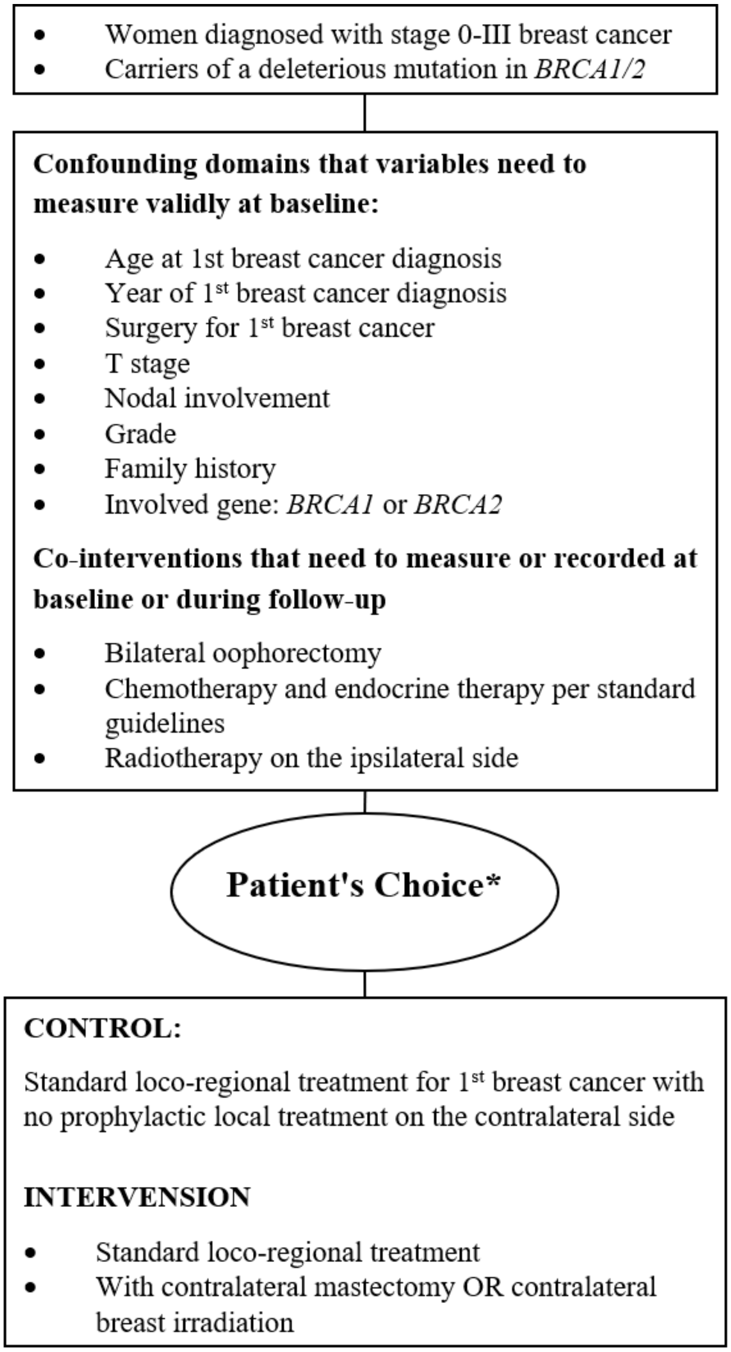

*The authors would like to emphasize that this trial is not randomized.

References in the Supplement:

1. van Sprundel TC, Schmidt MK, Rookus MA, Brohet R, van Asperen CJ, Rutgers EJ. Risk reduction of contralateral breast cancer and survival after contralateral prophylactic mastectomy in *BRCA1* or *BRCA2* mutation carriers. *Br J Cancer.* 2005;93(3):287-292.
2. Kiely BE, Jenkins MA, McKinley JM, et al. Contralateral risk-reducing mastectomy in *BRCA1* and *BRCA2* mutation carriers and other high-risk women in the Kathleen Cuningham Foundation Consortium for Research into Familial Breast Cancer (kConFab). *Breast Cancer Res Treat.* 2010;120(3):715-723.
3. Evans DG, Ingham SL, Baildam A, et al. Contralateral mastectomy improves survival in women with *BRCA1/2*-associated breast cancer. *Breast Cancer Res Treat.* 2013;140(1):135-142.
4. Metcalfe K, Gershman S, Ghadirian P, et al. Contralateral mastectomy and survival after breast cancer in carriers of *BRCA1* and *BRCA2* mutations: retrospective analysis. *BMJ.* 2014;348:g226.
5. Heemskerk-Gerritsen BA, Rookus MA, Aalfs CM, et al. Improved overall survival after contralateral risk-reducing mastectomy in *BRCA1/2* mutation carriers with a history of unilateral breast cancer: a prospective analysis. *Int J Cancer.* 2015;136(3):668-677.
6. Evron E, Ben-David AM, Goldberg H, et al. Prophylactic irradiation to the contralateral breast for *BRCA* mutation carriers with early-stage breast cancer. *Ann Oncol.* 2019;30(3):412-417.
